# Musical Expertise Increases Top–Down Modulation Over Hippocampal Activation during Familiarity Decisions

**DOI:** 10.3389/fnhum.2017.00472

**Published:** 2017-09-26

**Authors:** Pierre Gagnepain, Baptiste Fauvel, Béatrice Desgranges, Malo Gaubert, Fausto Viader, Francis Eustache, Mathilde Groussard, Hervé Platel

**Affiliations:** Normandie Université, UNICAEN, PSL Research University, EPHE, INSERM, U1077, CHU de Caen, Neuropsychologie et Imagerie de la Mémoire Humaine, Caen, France

**Keywords:** fMRI, semantic memory, hippocampus, effective connectivity, music

## Abstract

The hippocampus has classically been associated with episodic memory, but is sometimes also recruited during semantic memory tasks, especially for the skilled exploration of familiar information. Cognitive control mechanisms guiding semantic memory search may benefit from the set of cognitive processes at stake during musical training. Here, we examined using functional magnetic resonance imaging, whether musical expertise would promote the top–down control of the left inferior frontal gyrus (LIFG) over the generation of hippocampally based goal-directed thoughts mediating the familiarity judgment of proverbs and musical items. Analyses of behavioral data confirmed that musical experts more efficiently access familiar melodies than non-musicians although such increased ability did not transfer to verbal semantic memory. At the brain level, musical expertise specifically enhanced the recruitment of the hippocampus during semantic access to melodies, but not proverbs. Additionally, hippocampal activation contributed to speed of access to familiar melodies, but only in musicians. Critically, causal modeling of neural dynamics between LIFG and the hippocampus further showed that top–down excitatory regulation over the hippocampus during familiarity decision specifically increases with musical expertise – an effect that generalized across melodies and proverbs. At the local level, our data show that musical expertise modulates the online recruitment of hippocampal response to serve semantic memory retrieval of familiar melodies. The reconfiguration of memory network dynamics following musical training could constitute a promising framework to understand its ability to preserve brain functions.

## Introduction

Musical expertise enhances the functioning of brain networks responsible for auditory and motor processing (Fauvel et al., 2013). Recent research also suggest that such skills may also transfer to other domain of cognition such as language (Schellenberg and Weiss, 2013) given that they have a lot of features in common (Schön et al., 2010; Patel, 2011). Here, we examined whether musical training could benefit beyond sensory and motor domains and also promote dynamic interaction across brain systems underlying semantic memory access to melodies and verbal material such as proverbs.

When musicians have to rate their familiarity with a melody, hippocampus, as well as cortical midline structures, are activated more strongly compared with non-musicians (Groussard et al., 2010a). These regions overlap with the default network previously associated with spontaneous and self-generated thought (Andrews-Hanna et al., 2014). Yet, it is unclear how far the hippocampus may contribute to semantic decisions. On one hand, hippocampal activation during semantic decision might be merely incidental to the process of accessing memory, making no direct contribution to familiarity decision (*parallel activation hypothesis*), as proposed for instance by early models assuming independence of memory systems (e.g., Tulving, 1995). Indeed, a growing body of evidence suggests that the hippocampus is activated along with the semantic processing network during semantic decision tasks (e.g., Kapur et al., 1995; Leveroni et al., 2000; Bernard et al., 2004; Ryan et al., 2010). On the other hand, the hippocampus may directly influence familiarity processing (*top–down modulation hypothesis*; for a review on the interactions between the prefrontal cortex and hippocampus, see Rubin et al., 2017). For instance, the association of a concept with contextual autobiographical details has been proposed to make important contributions to the content and organization of semantic memory (Vallée-Tourangeau et al., 1998; Westmacott and Moscovitch, 2003).

From that latter perspective and considering the set of cognitive processes at stake during musical training, such as creative and self-generated thinking, which have been shown to benefit from goal-directed processing and cognitive control (Beaty et al., 2015), musical expertise would promote top–down influence during generation of hippocampally based goal-directed thoughts. Mounting evidence suggests that such top–down influences originate from the pars orbitalis (i.e., mainly Brodmann area, BA 47) in left inferior frontal gyrus (LIFG), a prominent hub of the semantic system sustaining the cognitive control processes that guide access to relevant information in semantic memory (Thompson-Schill et al., 1997; Wagner et al., 2001; Badre et al., 2005; Rodd et al., 2005; Badre and Wagner, 2007; Bedny et al., 2008; Whitney et al., 2009; Jefferies, 2013). Pars triangularis (i.e., mainly BA 45) in the LIFG would rather contribute to resolve competition among active representations following retrieval but does not directly participate to the top–down control of retrieved representations in memory (Badre and Wagner, 2007).

We used functional magnetic resonance imaging (fMRI) in musicians and non-musicians to examine whether hippocampal enhancement when accessing to familiar melodies may reflect the existence of increased control abilities following musical training. We intended to assess whether such hippocampal enhancement is promoted by the excitatory influence of the pars orbitalis in the LIFG to meet specific task demand (i.e., speed and accuracy), or alternatively simply arise from incidental reactivation. Moreover, our design also included French proverbs to assess whether or not such improvement in top-down regulation dynamics following musical practice might have transferred to other domains of semantic memory (Fauvel et al., 2013; Schellenberg and Weiss, 2013).

After characterizing the involvement of these two structures (LIFG and hippocampus) during familiarity decision and how their recruitment may interact with expertise and material, we used behavioral partial least squares (PLS) analyses to estimate their contribution to familiarity decision times. We then used dynamic causal modeling (DCM) to (1) test the hypothesis that the LIFG and hippocampus interact during familiarity decision (by comparing models that included modulation of the connection between these regions with others that did not), (2) assess whether hippocampal activation precedes or follows LIFG recruitment (by comparing models where neural activity is driven either by the LIFG or by the hippocampus), and (3) assess whether musical expertise may modulate the strength of top–down influence over hippocampus activity during semantic memory search.

## Materials and Methods

### Participants

Forty healthy right-handed adult participants were included in this study (age range: 20-35 years; male/female sex ratio: 20/20). Participants were divided into two groups, one comprising 20 musicians, and one enclosing 20 non-musicians. One non-musician participant was excluded because of uncomfortable positioning in the scanner that interrupted the experiment. The musicians were recruited from a music conservatory and private or voluntary music schools, where they had learned music theory for a minimum of 7 years. None of our musician participants had absolute pitch or were autodidact. They played a variety of instruments (violin, cello, guitar, flute, recorder, trumpet, clarinet, and piano). On average, they had begun their musical training at the age of 7.55 years (±1.87 years) and all stated that they were actively engaged in music at the time of the study [mean (*SD*) duration of uninterrupted music training: 15.3 (3.7) years; min = 8 years; max = 26 years]. Absence of formal musical training (except for basic music instruction at secondary school) was carefully verified in the non-musician group through a self-report questionnaire and an interview. However, they were normal listeners, as they reported enjoying listening to music regularly, and scored normally on a pitch perception test consisting in detecting subtle frequency shifts in melodies (*M* = 9.61/10 and *SD* = 0.68). None of the participants had any history of neurological or psychiatric disorders. They gave their written informed consent prior to taking part, and the research protocol was approved by the ethics committee.

The musician and non-musician participants did not differ significantly in terms of mean age (**Table 1**), *t*(37) = 1.55, *p* = 0.13, or sex ratio, χ^2^(1, *N* = 39) = 0.03, *p* = 0.87. However, non-musicians had significantly more years of education than musicians, *t*(37) = 2.25, *p* = 0.03, which could be explained presumably by the fact that in France musical studies and high musical diploma are generally not accounted as university graduation (and probably do not reflect than musicians are less educated).

**Table 1:** Mean (SD) demographic information.

	Musicians (*n* = 20)	Non-musicians (*n* = 19)	*t*/χ^2^ statistics	*p*-values
Age (years)	22.85 (3.05)	24.58 (3.91)	–1.55	0.13
Education (years)	15.15 (0.99)	16.32 (2.08)	–2.25	<0.05
Sex ratio (male/female)	10/10	10/9	0.03	0.87

### Task and Stimuli

**FIGURE 1:**
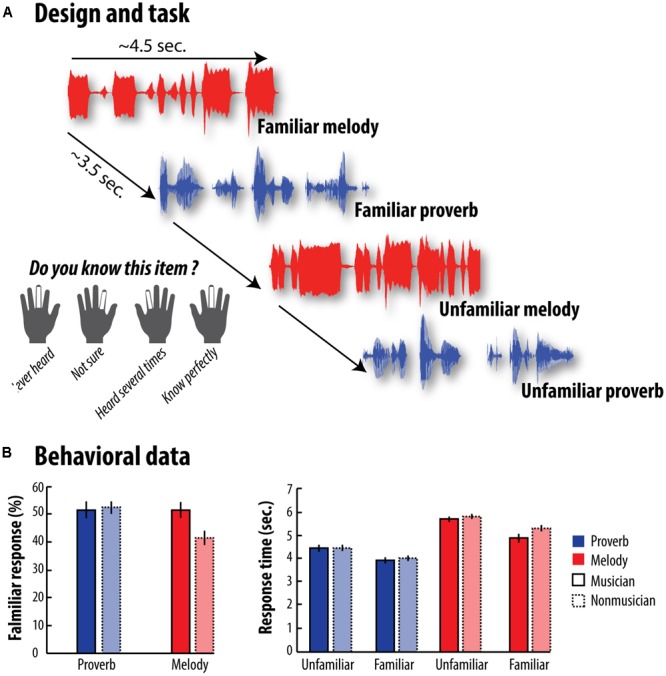
Design and Behavioral results - **(A)** We recorded participants’ brain activity while they performed the familiarity task. After listening either to melodies or to French proverbs, they had to rate their familiarity level on a 4-point scale by pressing buttons under their fingers. **(B)** The left-hand graph shows the percentage of French proverbs and melodies judged to be familiar by both musicians and non-musicians. The right-hand graph shows response times in each category for the two groups. Error bars represent the standard error of the mean (SEM). Statistical analyses are reported in details in the main text. Overall, musicians rated more melodies as familiar than non-musicians did (this difference was absent for French proverbs). Furthermore, we found that musicians judged more quickly melodies as familiar compared to non-musicians, while this effect was not observed for unfamiliar melodies or proverb in general.

Participants had to judge the level of familiarity of 60 excerpts of melodies and 60 excerpts of French proverbs using a 4-point scale. This task was specifically design to assess semantic memory using a familiarity decision. Participants were instructed to press the button 1 if they were sure that they never had heard the melody before (Fam1), the button 2 if they were not sure whether they had heard it before or not (Fam2), the button 3 if they knew having heard it several times (Fam3) and the button 4 if they knew it extremely well (Fam4) (**Figure 1A**). Stimuli consisted of 5-6-s excerpts more or less familiar and they were selected from a pilot study in which 79 adults (57 men and 22 women, age range: 18-30 years), including 25 musicians, had to rate the familiarity levels of 110 French proverbs and 80 melodies on this same 4-point familiarity scale. This initial selection ensured *a priori* that about the same number of items corresponded to each point of the familiarity scale, thus limiting any potential unbalance between familiar and unfamiliar items. From this initial piloting, we observed that 17/22 items were associated to the first anchor of the scale, 14/8 to the second, 15/12 to the third, and 14/18 to the fourth, for melodies and proverbs, respectively.

Melodies were taken from both the classical and modern repertoires, and were played on a digital keyboard using a flute voice without orchestration. French proverbs were recorded with a monotonous speech rate and a neutral voice intonation. Participants were instructed to give their response as soon as they feel really confident about the accuracy of their memory. They were further instructed that their response should occur after the onset of the stimulus but before the next one (i.e., corresponding to a time-window of approximately 8 s).

This initial selection ensured that about the same number of items corresponded to each point of the familiarity scale.

Participants underwent four fMRI scanning sessions, each divided into two tasks: a congruence judgment task (data not shown here; details of this experiment can be found in Groussard et al., 2010b), and the familiarity task (4-point familiarity scale) that we focused on in this study. The order of these tasks was counterbalanced across participants.

Before the fMRI recording began, participants practiced our tasks using items that were not presented in the main tasks. Items were played through MR-compatible headphones (Confon, Magdeburg, Germany) using an electrodynamic audio system that ensured attenuation of scanner noise up to 45 dB. The volume was slightly adjusted individually to ensure that each participant could hear the items clearly, above the noise of the MRI scanner. Items were presented using E-Prime software (Psychology Software Tools, Pittsburgh, PA, United States) implemented within IFIS System Manager (Invivo, Orlando, FL, United States). Participants were asked to close their eyes and maintain a high level of attention on the task.

Response times were cleaned up by excluding outlier data points two times above or below the median absolute deviation (MAD; Leys et al., 2013), computed separately for each condition. MAD is a robust measure of dispersion given by the following formula: MAD = b M_i_(|x_i_ - M_j_(x_j_)|), where x_j_ is the number of original observations, M_i_ the median, and *b* = 1.4826, a constant linked to the assumption of normality of the data, disregarding the abnormality induced by outliers (Leys et al., 2013). Based on this procedure, fewer than 10% of the items were discarded for each participant. A debriefing questionnaire took place immediately after the scanning sessions, mainly to ensure that nothing had disturbed the participants during the experiment.

### Imaging Acquisition Parameters

All images were acquired using the Philips (Eindhoven, The Netherlands) Achieva 3.0 T scanner. For each participant, a high-resolution T1-weighted anatomical image was first acquired using a 3D fast field-echo sequence (3D-T1-FFE sagittal; TR = 20 ms, TE = 4.6 ms, flip angle = 20, 170 slices, slice thickness = 1 mm, FOV = 256 mm × 256 mm, matrix = 256 × 256, acquisition voxel size = 1 mm × 1 mm × 1 mm), followed by a high-resolution T2-weighted anatomical image (2D-T2-SE sagittal; SENSE factor = 2, TR = 500 ms, TE = 80 ms, flip angle = 90°, 81 slices, slice thickness = 2 mm, FOV = 256 mm × 256 mm, matrix = 256 × 256, acquisition voxel size = 2 mm × 1 mm × 1 mm), and a non-EPI T2 star image (2D-T2 Star-FFE axial; SENSE factor = 2, TR = 3505 ms, TE = 30 ms, flip angle = 90°, 70 slices, slice thickness = 2 mm, FOV = 256 mm × 256 mm, matrix = 128 × 128, acquisition voxel size = 2 mm × 2 mm × 2 mm).

Functional data were acquired using an interleaved 2D T2 star EPI sequence designed to reduce geometric distortions and magnetic susceptibility artifacts (2D-T2 Star-FFE-EPI axial; SENSE factor = 2, TR = 2382 ms, TE = 30 ms, flip angle = 80°, 44 slices, slice thickness = 2.8 mm, matrix = 80 × 80, FOV = 224 mm × 224 mm, acquisition voxel size = 2.8 mm × 2.8 mm × 2.8 mm, 207 volumes per run). The functional volumes of the familiarity decision task were collected during two functional sessions.

### Univariate fMRI Data Analyses

Data were analyzed using statistical parametric mapping software (SPM8; Wellcome Trust Centre for Neuroimaging, London). During preprocessing, images were first corrected for slice acquisition delay, before being spatially realigned to correct for motion. In order to reduce geometric distortions, we used a methodology developed and validated by Villain et al. (2010), in which co-registration of the EPI volumes onto the T1 image was carried out in three steps: (1) the non-EPI T2 star volume was first co-registered onto the mean EPI image of the two runs; (2) the T2 image was then co-registered onto the co-registered non-EPI T2 star volume; and finally (3) the T1 volume was co-registered onto the co-registered T2 image. Images were then normalized using the parameters derived from the non-linear normalization of individual gray-matter T1 images to the T1 template of the Montreal Neurological Institute (MNI), and spatially smoothed using an 8-mm FWHM Gaussian kernel.

The preprocessed time series were concatenated across sessions to facilitate subsequent DCM analyses. Based on participants’ responses, items associated with the first two (Fam1 and Fam2) and last two (Fam3 and Fam4) points of the familiarity scale were aggregated to create an unfamiliar condition and a familiar condition. A general linear model (GLM) was estimated for each voxel, creating the regressors of interest by convolving a delta function modeled as a 4.5-s short epoch (median of all stimulus durations) at stimulus onset with a canonical hemodynamic response function (HRF) for each condition of interest (i.e., unfamiliar melody, unfamiliar French proverbs, familiar melody, familiar French proverbs). The regressors of no interest were the six realignment parameters, the sines and cosines of up to three cycles per run to capture low-frequency drifts, and constant terms to remove the mean of each run, as well as an additional regressor for items with no button press. Individual parameter estimates were then extracted and averaged in each region of interest (ROI), namely the left hippocampus and pars orbitalis of the LIFG, which were defined using the automatic anatomic labeling (AAL) atlas (Tzourio-Mazoyer et al., 2002). We only consider left hippocampus and LIFG in our analysis, firstly in coherence with the neuroimaging literature showing prominent left side activation for familiarity and semantic memory processes for verbal and musical stimuli (Platel et al., 2003; Binder et al., 2009), secondly considering that the activation results obtained both by musicians and non-musicians participants for our verbal and musical semantic task were clearly left lateralized (Groussard et al., 2010a). Additional voxel-based analyses were performed by entering first-level activation maps for each condition of interest into flexible analyses of variance (ANOVAs) implemented in SPM, which used pooled error and correction for non-sphericity to create *t* statistics. The SPMs were thresholded for voxels whose statistics exceeded a peak threshold set at *p* = 0.05 family wise error (FWE) corrected across the whole brain or within the appropriate search volumes of interest, using random field theory.

### Behavioral PLS Correlation Analyses

We conducted behavioral PLS analyses to understand the relationship between the neural responses and familiarity decision times for familiar items across the hippocampus voxels composing our ROI (McIntosh and Lobaugh, 2004; Krishnan et al., 2011; Abdi and Williams, 2013). Behavioral PLS is a multivariate technique that reduces a set of voxels (i.e., variables) to a ranked series of independent latent variables (LVs) expressing the greatest possible covariance (or correlation) with behavioral scores. Voxel activity first has to be aligned and stacked across participants into a brain activation matrix (X) of *J* rows (i.e., participants) and *N* voxels. Normalized brain images are therefore used for this purpose. Here, we restricted our analysis to the left hippocampus. Within this mask, parameter estimates for familiar melodies and French proverbs were extracted for each voxel, and the resulting vector of voxels was stacked across participants (separately for musicians and non-musicians). Familiarity decision times for familiar items were encoded in a *J* rows (i.e., participants) matrix (Y). Both Y and X data tables were then mean-centered and rescaled so that their norm (i.e., square root of the sum of squares) was equal to one, as is usually done during PLS analysis (see Krishnan et al., 2011; Abdi and Williams, 2013). The cross-block product of X and Y (i.e., Y^T^X) therefore produced a 1 × *N* voxels correlation matrix (R) encoding the relationship between each voxel and familiarity decision times across participants. The singular value decomposition (SVD) was then applied to this R correlation matrix, such that R was decomposed into three matrices (R = UΔV^T^), where U was the matrix of behavioral saliences and V voxel saliences. The SVD identified the LVs that maximized the covariance between voxel activation (X) and behavioral scores (Y). Each LV in V contained a spatial pattern depicting the brain regions where the activation showed the strongest relation to our behavioral scores. The brain scores (X^T^V) reflected the summed contribution of each participant’s expression of a particular LV pattern. Correlations between participants’ brain scores and behavioral variables therefore indicated how each LV optimally represented relations between behavior and brain activity. The statistical significance of the LVs was assessed using 5000 permutation tests, during which participants’ brain data matrices were randomly reassigned to behavioral scores and a singular value was recomputed each time. The number of times a singular value exceeded the original singular value indicated the probability of significance of the original LVs (McIntosh et al., 1996). To compute the significance of voxel salience, we applied bootstrapping with replacement, and recomputed the SVD for each new bootstrap sample. During resampling, LV axes may rotate and change the sign of saliences during SVD computation. Procruste rotation was therefore applied to each new sample to correct for these inversions and return it to the original sample space (Milan and Whittaker, 1995). This procedure yielded a bootstrap distribution of voxel saliences that could then be transformed into a *z*-score (by dividing initial voxel salience by the standard error of the bootstrapped distribution) to assess the significance of a given voxel (McIntosh and Lobaugh, 2004). It should be noted that this multivariate technique quantifies the relationship between a voxel and a given dimension, and is performed in a single analytic step. It therefore does not require correction for multiple comparisons across voxels, as is the case with multiple univariate independent testing (McIntosh and Lobaugh, 2004).

### DCM Analyses

Here, we used DCM (Friston et al., 2003) and Bayesian model selection (BMS; Penny et al., 2010) to assess (1) whether the LIFG and hippocampus interacted when a familiar item was identified, and (2) which region (LIFG or hippocampus) was activated first during semantic retrieval and drove activity in the network, and (3) assess whether musical expertise may modulate the strength of top–down influence over hippocampus activity during semantic memory search. After estimating the neural (and hemodynamic) parameters, BMS was used to select the preferred model at the group level, treating the optimum model across participants as a random effect.

The first eigenvariate time series for DCM analyses were extracted as follows: for each participant, we identified the maximum peak within the space defined by ROI binary masks using the familiar > unfamiliar contrast. An in-house program was then used to select the most significant contiguous voxels from this peak, corresponding to 5% of the total mask size, and create a new mask respecting anatomical demarcation. We then extracted the first eigenvariate from this new mask, and adjusted it for effects of no interest (i.e., the six realignment parameters, sines and cosines of up to three cycles per run to capture low-frequency drifts, and constant terms to remove the mean of each run).

We created 12 DCM models (for an illustration of the model space, see **Figure 4**). All models had bidirectional connections between the hippocampus and LIFG. These 12 models could be divided into two families of models. The first model family (i.e., input family) divided the model space into three subgroups, according to the source of the driving input. In the first subgroup, familiar and unfamiliar trials (aggregated across melodies and French proverbs) entered the system separately in the LIFG. In the second subgroup, familiar and unfamiliar trials entered the system in the left hippocampus. In the third subgroup, familiar and unfamiliar trials entered the system in both the LIFG and the left hippocampus. The second model family divided the model space into four subgroups that differed according to whether or not the intrinsic connections were additionally modulated by familiar trials (modeled separately here for melodies and French proverbs). In the first subgroup, models included bottom–up modulation of the connection from the hippocampus to the LIFG during familiar trials. In the second subgroup, this modulation was top–down, and in the third one it was bidirectional. Finally, the fourth subgroup of models did not include any additional modulation. After estimating all 12 models for each participant, we performed the group BMS as implemented in SPM 12 (version DCM 12 revision 5729). This produces the exceedance probability (EP; i.e., extent to which each model is more likely than all the other models) and expected posterior probability (EPP; i.e., probability of a model generating the observed data).

## Results

### Musical Expertise Increases Access to Familiar Melodies but Not Familiar Proverbs

In our first analysis, we investigated whether the percentage of items judged to be familiar depended on the type of material (melodies vs. French proverbs), and/or on the participants’ expertise (musicians vs. non-musicians). An ANOVA including expertise as a between-participants factor indicated a significant effect of material, with more French proverbs being judged as familiar than melodies, *F*(1,37) = 9.32, *p* = 0.004, η^2^ = 0.20, and a significant Material × Expertise interaction, *F*(1,37) = 8.49, *p* = 0.006, η^2^ = 0.19. Further *a priori* comparisons showed a significant effect of expertise (i.e., musicians > non-musicians) for melodies, *t*(37) = 2.94, *p* = 0.0028, η^2^ = 0.07, but not for French proverbs, *t*(37) = -0.30, *p* = 0.38, η^2^ = 0.008.

In our second analysis, we tested whether participants’ familiarity decision times depended on familiarity (familiar vs. unfamiliar), material (melodies vs. French proverbs), and/or expertise (musicians vs. non-musicians). This ANOVA revealed a significant Material x Familiarity interaction indicating that the familiarity effect was stronger for melodies than for French proverbs across groups, *F*(1,37) = 6.73, *p* = 0.014, η^2^ = 0.15 although both melodies, *F*(1,37) = 61.52, *p* = 2.2 × 10^-9^, η^2^ = 0.62 and French proverbs, *F*(1,37) = 73.21, *p* = 2.7 × 10^-10^, η^2^ = 0.66 were associated with significantly faster reaction times for familiar versus unfamiliar items. Material × Familiarity × Expertise interaction was not significant *F*(1,37) = 2.95, *p* = 0.094, η^2^ = 0.07. Interestingly, however, further *a priori* comparisons showed a significant Familiarity × Expertise interaction for melodies, *t*(37) = 1.81, *p* = 0.039, η^2^ = 0.05. This interaction was mainly driven by a significant difference between musicians and non-musicians for familiar melodies, *t*(37) = 2.05, *p* = 0.024, η^2^ = 0.05, which was absent for unfamiliar ones, *t*(37) = 1.22, *p* = 0.12, η^2^ = 0.03. This Familiarity × Expertise interaction was absent for proverbs, *t*(37) = 0.62, *p* = 0.24, η^2^ = 0.02, suggesting that musical skills does not transfer to verbal memory at least in our task and at the behavioral level. Taken together, these findings confirm that familiarity decision for melodies, as well as speed of access to stored representations, increase with musical expertise. The results of these behavioral analyses are reported in detail in **Figure 1B**.

### Hippocampal Responses Increase with Musical Expertise during Semantic Access to Familiar Melodies But Not Familiar Proverbs

To confirm the engagement of a left-lateralized network for retrieving familiar items, as observed in previous studies (Platel et al., 1997, 2003; Groussard et al., 2010b), we contrasted familiar and unfamiliar trials collapsed across both types of material (*p*_FWE_ < 0.05). Consistent with previous findings, we observed more activation for familiar items than for unfamiliar ones in a broad left-lateralized network. Two large LIFG clusters, centered on the pars orbitalis (*x* = -34, *y* = 30, *z* = -20; *z*_max_ = Inf, *p*_FWE_ = 4.4 × 10^-16^) and pars triangularis (*x* = -40, *y* = 42, *z* = 4; *z*_max_ = 7.59, *p*_FWE_ = 1.58 × 10^-14^), and previously involved in semantic memory retrieval, survived whole-brain cluster correction (for full whole-brain analyses, see Supplementary Table S1). Critically, when the search volume was restricted to left hippocampus, significant increase was also observed for familiar item in this structure (*x* = -16, *y* = -4, z = -12; *z*_max_ = Inf, *p*_FWE_ = 4.4 × 10^-16^). Overall, the familiarity effect was nonetheless stronger for melodies than for French proverbs. This was confirmed by the presence of a Material x Familiarity interaction that yielded significant differences both when the search volume was restricted to the pars orbitalis of the LIFG (*x* = -36, *y* = 28, *z* = -4; *z*_max_ = 6.67, *p*_FWE_ = 1.53 × 10^-8^), and left hippocampus (*x* = -14, *y* = -4, *z* = -14; *z*_max_ = 4.97, *p*_FWE_ = 1.17 × 10^-4^). Although this interaction was independent of expertise when the search volume was restricted to the pars orbitalis of the LIFG, it was stronger for musicians in the left hippocampus which showed an Expertise × Material × Familiarity interaction (*x* = -20, *y* = -16, *z* = -16; *z*_max_ = 4.55, *p*_FWE_ = 7.53 × 10^-4^).

**FIGURE 2:**
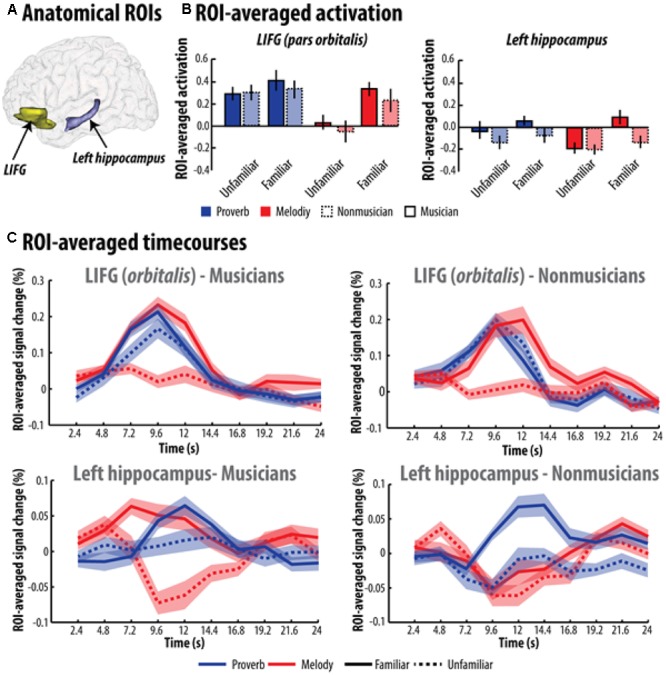
Region of interest (ROI) activity observed while musicians and non-musicians decided whether the melodies or French proverbs were familiar or not – **(A)** Illustration of anatomical ROIs corresponding to the LIFG/orbitalis and left hippocampus. **(B)** Averaged parameter estimates of the canonical HRF and **(C)** FIR estimation across time extracted within the orbitalis part of the LIFG and the left hippocampus while both groups listened to French proverbs and melodies. Error bars represent the standard error of the mean (SEM). Familiarity enhancement was stronger overall for melodies. However, there was a difference between familiar and unfamiliar melodies in the LIFG for both musicians and non-musicians, whereas this difference was only observed in the left hippocampus for musicians.

To confirm these findings, further ROI averaging analyses were conducted in the LIFG and left hippocampus (see **Figure 2A**). In the first series of ROI analyses, we extracted and averaged parameter estimates of the canonical HRF (see **Figure 2B**). Material x Familiarity ANOVAs, including expertise as a between-participants factor, showed a significant main effect of familiarity in both ROIs [pars orbitalis of the LIFG: *F*(1,37) = 94.4, *p* = 1 × 10^-11^, η^2^ = 0.72; left hippocampus: *F*(1,37) = 62.1, *p* = 2 × 10^-9^, η^2^ = 0.63]. Planned comparisons confirmed that activation was greater for familiar versus unfamiliar items for both types of material in the pars orbitalis [melodies: *F*(1,37) = 107.96, *p* = 1.6 × 10^-12^, η^2^ = 0.74; French proverbs: *F*(1,37) = 10.9, *p* = 0.0021, η^2^ = 0.23] as well as in the left hippocampus [melodies: *F*(1,37) = 35.14, *p* = 7.9 × 10^-7^, η^2^ = 0.17; French proverbs: *F*(1,37) = 31.7, *p* = 2 × 10^-6^, η^2^ = 0.46]. However, significant Material × Familiarity interactions were also observed in the pars orbitalis of the LIFG, *F*(1,37) = 29.96, *p* = 3.24 × 10^-6^, η^2^ = 0.45, as well as in the left hippocampus, *F*(1,37) = 6.28, *p* = 0.017, η^2^ = 0.14, indicating that the increase observed for familiar versus unfamiliar items was greater for melodies than for French proverbs. This interaction did not vary according to expertise in the pars orbitalis (all *F*s < 0.45), but was significantly more pronounced for musicians in the left hippocampus (i.e., an Expertise × Material × Familiarity interaction), *F*(1,37) = 9.6, *p* = 0.0037, η^2^ = 0.21. Results of these ROI analyses are reported in detail in **Figure 2B**.

We also extracted finite impulse response (FIR) timecourses using the MarsBarR toolbox in the second series of ROI analyses (see **Figure 2C**). Activity at each timepoint around the peak of the BOLD response (4.8-16.8 s) was extracted and entered into a Material × Familiarity × Timepoint ANOVA, including expertise as a between-participants factor. This additional analysis confirmed that both the pars orbitalis, *F*(1,37) = 16.36, *p* = 0.00026, η^2^ = 0.31 and the left hippocampus, *F*(1,37) = 11.04, *p* = 0.002, η^2^ = 0.23, were associated with greater activation during familiar melodies than unfamiliar ones in musicians. The same pattern was observed for non-musicians in the pars orbitalis, *F*(1,37) = 9.84, *p* = 0.0033, η^2^ = 0.21, though not in the hippocampus, *F*(1,37) = 0.17, *p* = 0.68, η^2^ = 0.004. Moreover, whereas we found a difference between familiar and unfamiliar French proverbs in the non-musicians’ hippocampus, *F*(1,37) = 8.63, *p* = 0.0057, η^2^ = 0.19, this effect was non-significant for musicians, *F*(1,37) = 0.45, *p* = 0.506, η^2^ = 0.01, and absent in the LIFG for both groups (all *F*s < 0.62). These distinctions were confirmed by the presence of a significant Expertise × Familiarity × Material interaction in the left hippocampus, *F*(1,37) = 6.58, *p* = 0.014, η^2^ = 0.15, which was not observed in the LIFG, *F*(1,37) = 0.29, *p* = 0.59, η^2^ = 0.008.

Taken together, these findings show that accessing stored memories of familiar melodies, as opposed to unfamiliar ones, activates the pars orbitalis of the LIFG and the left hippocampus. This pattern was consistent across both types of ROI analyses (parameter estimates of the canonical HRF and FIR), which further confirmed that this difference (familiar versus unfamiliar melodies) was clearly stronger than the difference in activation between familiar and unfamiliar proverbs in both the LIFG and the left hippocampus. This suggests that melodies are more powerful cues for triggering retrieval processes and access to representations stored in memory in general. Alternatively, if the identification point of familiar French proverbs is time-locked to specific words in a sentence, semantic traces encoding familiar French proverbs may be less tractable when the stimulus function is modeled as short epochs.

Interestingly, however, this Material × Familiarity interaction observed in both structures also interact with expertise but only in the left hippocampus. This three-way interaction was characterized by the fact that only musicians activate the left hippocampus for familiar melodies, while no difference between familiar and unfamiliar melodies was observed in the left hippocampus of non-musicians. Both groups did show a difference in activation between familiar versus unfamiliar French proverbs in the left hippocampus, confirming that expertise plays an important role in the recruitment of the hippocampus during access to semantic memory.

### Activation of the Hippocampus Is Linked to Familiarity Decision Times for Melodies in Musicians But Not in Non-musicians

**FIGURE 3:**
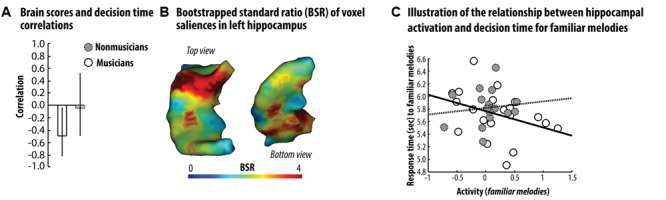
Results of the behavioral PLS correlation analyses in the left hippocampus *–* **(A)** Correlation coefficients between the first LV brain scores and familiarity decision times showed that there was a significant negative relationship between brain response to familiar melodies and decision times in musicians (in white/plain line), but not in non-musicians (gray/dashed line). Bars represent bootstrapped 95% CI. **(B)** Voxel salience associated with the first LV showed that this significant relationship was present in voxels mainly localized in the anterior section of the hippocampus. **(C)** Scatterplots illustrating the relationship between decision time and the neural response to familiar melodies averaged across voxel associated with a positive and significant salience. Taking together, these data show that semantic decision time during processing of familiar melodies accelerates as neural response in the anterior section of the hippocampus increases. However, such relationship is specifically observed in musicians and is absent in participants without musical expertise.

We next examined whether participants’ ability to recruit the left hippocampus was related to their familiarity decision times. We used PLS correlation analyses to study the relationship between activation during familiar trials in the LIFG and left hippocampus, and familiarity decision times (see Materials and Method section for details about this analysis). Our analysis was restricted to a single mask corresponding to the left hippocampus. For proverbs, the first LV accounted for 70% of the cross-table covariance between brain activation and decision times, and was not significantly different from random noise, as assessed by permutation testing (*p* = 0.12). This finding does not support the existence of a relationship between hippocampal response to familiar proverb and semantic decision time. For melodies, the first LV accounted for 78% of the cross-table covariance between brain activation and decision times, and was significantly different from random noise, as assessed by permutation testing (*p* < 0.05). Critically, the first LV brain scores correlated significantly and negatively with decision times for musicians (*r* = -0.46, bootstrapped 95% Confidence Interval (CI) [-0.78, -0.05]; see **Figure 3A**), whereas this relationship was not significant in non-musicians (*r* = -0.08, bootstrapped 95% CI [-0.48, 0.56]; see **Figure 3A**). Most of the voxels associated with a positively significant salience (assessed by means of bootstrapping; see **Figure 3B**) were localized within the anterior section of the hippocampus. These findings showed that in musicians, familiarity decision times for familiar melodies accelerated as upregulation of the left hippocampus increased (see **Figure 3C**). Interestingly, the correlation between LV brain scores and reaction times indicated a non-significant relationship in non-musicians (see **Figures 3A,B**). The selection of familiar melody traces may have been less efficient in non-musicians, and increased left hippocampus search time, may have compromised familiarity decision speed in order to preserve accuracy. These findings thus reveal that enhancement of the left hippocampal response during familiar melody processing is correlated with faster familiarity decision times when the degree of prior knowledge and expertise is strong enough.

### Top–Down Signal between the LIFG and Left Hippocampus Increases with Musical Expertise for Both Familiar Melodies and Familiar Proverbs

Our findings have so far shown that the left hippocampus is not just incidentally co-activated with the LIFG during memory operations, but is also linked to familiarity decision times. The presence of greatly enhanced hippocampal activity for familiar melodies, combined with upregulation of the LIFG response during familiarity decision, is consistent with the notion that a prefrontal network, involving the LIFG, triggers a signal to enhance the response of the left hippocampus, whose activity contributes to familiarity decision operations. However, we had not yet to formally demonstrate any causal link between LIFG and hippocampal activity during memory retrieval. One possibility was that hippocampal representations are activated in parallel, but do not interact with the memory processes engaged by the LIFG. Alternatively, top–down mechanisms mediated by the LIFG might actively enhance hippocampal activity during familiar trials. To assess these hypotheses, we used DCM and BMS to test whether the LIFG down-regulated activity in the left hippocampus during familiar items.

**FIGURE 4:**
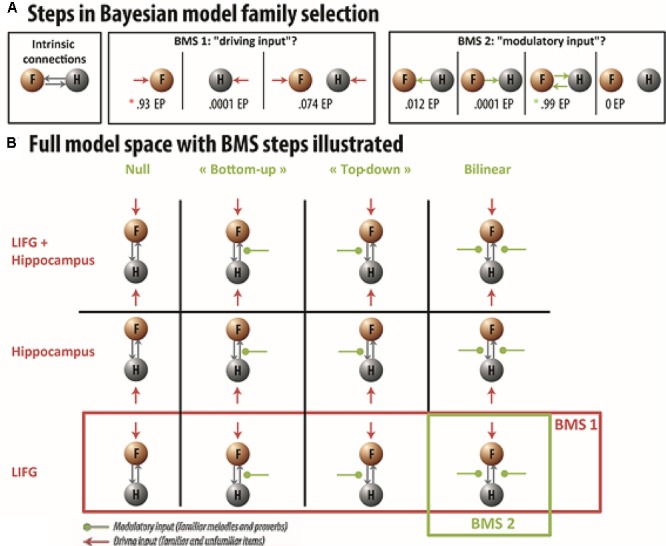
Dynamic causal modeling (DCM) model space and BMS procedure *–* **(A)** Full intrinsic connections between the LIFG (F) and hippocampus (H) were specified for all competing models. We then compared three driving input families, where activity entered the model either in the hippocampus, the LIFG, or both. Within the winning driving input family, we then compared which modulatory input was more likely: top–down, bottom–up, bidirectional or null. **(B)** Illustration of the entire model space with BMS steps. Gray arrows represent the intrinsic connections between the nodes. Red arrows correspond to the driving input of the model. Green arrows illustrate the presence of a modulatory input for familiar melodies and French proverbs on connection strength. The LIFG driving input family won [indicated by the red asterisk in **(A)** and the red rectangle in **(B)**] with an EP of 0.93. Within the LIFG driving input family, the model with a bidirectional modulatory input won the second BMS with an EP of 0.99 [indicated by the green asterisk in **(A)** and the green rectangle in **(B)**].

To test the hypothesis that the LIFG caused the increased activation in the left hippocampus during familiar trials, we first tested whether the familiarity enhancement started from the LIFG, by comparing models within the first input family distinction (input could enter either the LIFG, the left hippocampus, or both; see **Figure 4**). This analysis overwhelmingly favored models in which driving inputs entered the LIFG (*EP* = 0.93, *EPP* = 0.55) over models in which inputs entered either the left hippocampus (*EP* = 0.0001, *EPP* = 0.074) or both the LIFG and the left hippocampus (*EP* = 0.14, *EPP* = 0.31). *EP* refers to the extent to which one model is more likely than the other models being considered, whereas *EPP* is the probability of a model generating the observed data. We then restricted our model space to models in which the driving input originated from the LIFG, and compared the remaining models within the second modularity family distinction. These driving inputs were meant to represent the influence of memory retrieval in the network. Thus, for null models, activation differences across familiar and unfamiliar items in the hippocampus could solely be explained in terms of driving input, without further modulation of activity between the LIFG and hippocampus. For models including top–down or bottom–up modulation, activation differences across conditions in the hippocampus could also be explained by additional modulatory forces exerted on LIFG-hippocampus or hippocampus-LIFG connections. This analysis massively favored models including bidirectional (top–down and bottom–up) modulation (*EP* = 0.99, *EPP* = 0.65), over null (*EP* = 0, *EPP* = 0.04), top–down (*EP* = 0.0001, *EPP* = 0.08), and bottom–up (*EP* = 0.012, *EPP* = 0.24) models. Thus, our data provided strong evidence that hippocampal enhancement during familiarity decision is associated with modulatory signals from the LIFG (together with a bottom–up influence), and suggested that hippocampal recruitment is modulated online during the retrieval of memory traces.

The maximum *a posteriori* (MAP) estimates (parameters) of the intrinsic connections (DCM.A matrix) and modulatory inputs (DCM.B matrix) for familiar melodies and French proverbs were extracted from the winning model for each participant. Intrinsic (DCM.A) and coupling (DCM.B) parameters were summed to reflect the scaling induced by familiar stimuli on intrinsic connectivity (i.e., average connectivity in the absence of any experimental manipulation; see **Table 2**). At the group level, the modulation parameters (DCM.A + DCM.B matrices) thus obtained were entered in a series of one-sample *t*-tests, which confirmed that they were all significantly different from zero (all *p*s < 0.05). For both types of material, we found statistically significant coupling during familiar trials, which was positive from the LIFG to the left hippocampus (mean = 0.67), but negative from the left hippocampus to the LIFG (mean = -0.38). This indicated that the familiarity-related modulation regulating activity from the LIFG to the left hippocampus was excitatory, and that the left hippocampus sent an inhibitory modulatory signal back to the LIFG.

**Table 2:** Mean (SD) parameter estimates of DCM.A + B matrices in musicians and non-musicians for both types of material.

	Top–down (LIFG → Hipp)		Bottom–up (Hipp → LIFG)	
	Melodies	French expressions	Melodies	French expressions
Musicians	1.1 (0.61)	0.87 (0.51)	–0.46 (0.63)	–0.47 (0.66)
Non-musicians	0.31 (0.46)	0.37 (0.37)	–0.26 (1.06)	–0.31 (0.79)

These modulation parameters were also integrated into a Direction (top–down vs. bottom–up) × Material ANOVA where expertise was the between-participants factor. Results indicated that parameter values differed significantly between the top–down and bottom-up modulations, *F*(1,37) = 83.25, *p* = 5.2 × 10^-11^, η^2^ = 0.69. Critically, we found a significant Direction × Expertise interaction, *F*(1,37) = 13.12, *p* = 0.0009, η^2^ = 0.26, which showed that musicians’ top–down parameters were higher than those of non-musicians, *F*(1,37) = 22.17, *p* = 0.00004, η^2^ = 0.37, while bottom–up parameters did not change with expertise, *F*(1,37) = 0.82, *p* = 0.36, η^2^ = 0.02 (see **Table 2**). This interaction, however, did not vary with material (*F* < 2.75). It should be noted that in DCM, between-group comparisons on estimated parameters can only be performed within the same winning model or family (see Seghier et al., 2010). However, to confirm that additional top–down modulation only occurred in musicians, we also performed separate BMSs on each group. These revealed that musicians overwhelmingly favored models featuring bidirectional modulation (*EP* = 0.91, *EPP* = 0.52), while the family that only included bottom–up modulation was slightly preferred in non-musicians (*EP* = 0.7, *EPP* = 0.46).

## Discussion

We assessed whether musical expertise increases hippocampal engagement during a familiarity decision task on both melodies and French proverbs by promoting top–down frontal mechanism that guides access to relevant information in semantic memory. Analyses of behavioral data confirmed that familiarity decision for melodies, as well as speed of access to stored representations, increase with musical expertise. However, we failed to find that musical skills transfer to the ability to access semantic memory for verbal proverbs. Univariate voxel-based and ROI analyses confirmed the involvement of the pars orbitalis of the LIFG and the left hippocampus in processing familiar versus unfamiliar items. In addition, this analysis showed that musicians had greater left hippocampal activation than non-musicians during the processing of incoming familiar melodies compared to unfamiliar ones, an effect not observed for proverbs. We then examined the relationship between participants’ left hippocampus recruitment and their familiarity decision times through a behavioral PLS correlation analysis. Again, the results were different for musicians and non-musicians, as decision time for familiar melodies only accelerated with left hippocampal neural up-regulation in musicians. Finally, we used DCM analysis to investigate the neural dynamics between the LIFG and left hippocampus during familiarity decision. This analysis revealed the existence of an excitatory top–down signal that was sent from the LIFG to the left hippocampus in the familiar condition. Moreover, further BMS and model-estimated parameter analyses revealed that this excitatory signal was critically stronger in musicians than in non-musicians, but not different between melodies and proverbs.

Taken together, these results unambiguously reinforce our hypothesis that musicians can exert a control over hippocampal representations to serve musical familiarity decision. Such top–down control over the hippocampus during semantic access does not happen in non-musicians who have a lower level of pre-existing knowledge and experiences with melodies. The nature of the hippocampal contribution remains unclear, however. Previous studies had shown that the hippocampus is activated during semantic memory tasks (Leveroni et al., 2000; Bernard et al., 2004; Plailly et al., 2007), notably when these involve relational (Ryan et al., 2010), spatiotemporal (Maguire and Mummery, 1999), or personally relevant components attached to autobiographical and episodic details (Maguire and Mummery, 1999). Our data might therefore reflect the existence in musicians of increased activation of episodic details such as the spatiotemporal context or other self-relevant knowledge linked to hippocampal representations. Alternatively, however, the hippocampus could have a direct mechanistic function in processing familiar sounds. Teki et al. (2012) and Groussard et al. (2014) show that gray-matter volume in the hippocampus increases respectively with years of tuning experience in professional musicians (controlling for musical ability) and years of musical practice, suggesting that skilled exploration of melodies may rely on a consolidated template dependent upon the hippocampus. Given this structure’s well-known role in the binding of temporally adjacent events (Olsen et al., 2012), hippocampal representations during melody processing may help maintain temporal continuity during the playing of musical notes (Burunat et al., 2014) or predict incoming sounds based on pre-existing representations (e.g., Gagnepain et al., 2012). Indeed, previous studies using musical tasks have already shown that the hippocampus contribute to the extraction statistical regularities across complex acoustic patterns (Barascud et al., 2016) and to the binding of lyrics to melodies (Alonso et al., 2016).

Although our data did not allow us to disentangle these two alternative interpretations (episodic vs. auditory functional role), behavioral PLS correlation analyses further underscored the critical role of the left hippocampus in musical familiarity decision. The strength of left hippocampal activation to familiar melodies in musicians was related to the speed increase in familiarity decision. Interestingly, this relationship was absent for the non-musicians. These findings are consistent with the idea that hippocampally based representations consolidated in an experience-dependent manner (i.e., related to autobiographical details or, alternatively, to some forms of the auditory stream’s processing template) accelerate access to stored semantic memories. In this case, musicians’ distinctive memory traces would guide their choice in a swift and efficient manner. Conversely, non-musicians’ memory representations associated with music would be more general increasing attentional demand toward accuracy at the cost of memory search speed.

Moreover, PLS analyses further identified that this relationship exclusively observed in musicians was preferentially supported by the anterior portion of the left hippocampus. This finding echoed with previous results showing that musical expertise increases the volume of the anterior hippocampus (Groussard et al., 2010a, 2014), but also improves the sensitivity of this structure to the temporal mismatch of regular sound patterns (Herdener et al., 2010). Taking together, these data might thus support recent proposals implying a functional specialization of the anterior hippocampus toward pattern completion of coarse representations (Poppenk et al., 2013) and suggest an interesting emerging property of the anterior hippocampus with respect to musical expertise.

In accordance with our previous results (Groussard et al., 2010a) and other studies (Platel et al., 2003; Binder et al., 2009), showing prominent left side activation for familiarity and semantic memory processes for verbal and musical stimuli, we have not considered right-sided hippocampus and IFG modulation. However, right-sided structures play an important role to control semantic retrieval (e.g., Hallam et al., 2016) and musical expertise surely also modulates aspects of these networks given the set of skills promoted by musical training (e.g., Pallesen et al., 2010). Further works would be needed to identify the neural computations at stake across hemispheres and how musical expertise may modulate inter-hemispheric cooperation during memory retrieval.

Although the evidence described above was consistent with the notion that the LIFG sends a signal to enhance left hippocampal recruitment during familiarity decisions, the next step of the present study was to formally demonstrate that this recruitment is directly coordinated by prefrontal mechanisms to further guide access to memory representations. In agreement with this idea, DCM analyses performed in a network comprising the pars orbitalis of the LIFG and the left hippocampus revealed that neural activity was initiated in the LIFG, and the intrinsic coupling between the LIFG and the left hippocampus was further modulated by familiarity (e.g., with excitatory and inhibitory influences for top–down and bottom–up connections, respectively). The fact that neural activity was primarily driven by the LIFG suggests that hippocampal recruitment during familiarity decisions depends on controlled processing. The presence of a top–down excitatory signal emphasized the importance of the joint contribution of the LIFG and left hippocampus to probing memory representations during familiarity decisions. Interestingly, the inhibitory signal sent by the hippocampus to the LIFG suggests that the left hippocampus also modulates the dynamics of semantic memory access as the sounds unfold. Thus, our data provide evidence that hippocampal activity during memory retrieval is influenced by modulatory signals from the pars orbitalis. Interestingly, the strength of top–down modulation was stronger in musicians than in non-musicians while no difference was found for bottom–up parameters. This result suggests that musical expertise specifically increase top–down control during memory search. Confirming this finding, the separate BMSs performed for each group revealed that top–down modulation in fact only took place in musicians (the non-musicians’ winning model only featured bottom–up modulation). Moreover, we did not find that this increase in the top–down excitatory signal varied with the type of material, suggesting that the benefit of gaining control over hippocampal recruitment following musical expertise transfer to other modalities including languages. Although such transfer does not seem to produce any gain in performances in term of verbal memory abilities, it may explain why sometimes musical expertise is associated with better cognitive outcome following brain damage (Omigie and Samson, 2014) or might reduce deleterious effect of aging (Fauvel et al., 2013).

Finally, our findings bring fresh arguments to support the view that the role of the hippocampus in human cognition is not limited to episodic memory (Duff and Brown-Schmidt, 2012; Olsen et al., 2012). Our findings add to the growing body of evidence that the hippocampus can be activated in a controlled fashion along with the semantic processing network during semantic retrieval or semantic decision tasks (Kapur et al., 1995; Leveroni et al., 2000; Bernard et al., 2004; Plailly et al., 2007). Familiarity judgment and recognition of melodies is not, however, purely a semantic processing. Structural and perceptual information may also contribute to familiarity decision and recognition, at least to some extent, as hypothesized in various recognition model of faces (Bruce and Young, 1986), objects (Humphreys and Riddoch, 1987), or melodies (Peretz et al., 1994). However, we do emphasize the semantic aspects of our task given that: (1) our main findings focus around the LIFG, a critical structure supporting access and selection in semantic memory (Thompson-Schill et al., 1997; Wagner et al., 2001; Badre et al., 2005; Rodd et al., 2005; Badre and Wagner, 2007; Bedny et al., 2008; Whitney et al., 2009; Jefferies, 2013), and (2) the scale and instructions encouraged participants to rely on semantic attributes or properties of the melodies to decide whether or not they were highly familiar with them.

Our data indicate that hippocampus-related memory representations are not just merely incidental to the process of accessing semantic memory but can even contribute to semantic decisions (Vallée-Tourangeau et al., 1998; Westmacott and Moscovitch, 2003). This suggests that common representations and processes can contribute to both episodic and semantic memory (Lundstrom, 2003; Lundstrom et al., 2005; Rajah and McIntosh, 2005; Wais et al., 2006; Burianova and Grady, 2007), and that memory systems are more interdependent than previously thought (Henson and Gagnepain, 2010). Our data highlight that musical expertise is critical to the reconfiguration of regulatory dynamics between these interdependent memory networks, thus constituting a promising neurobiological framework to further apprehend how musical expertise-dependent plasticity may transfer to other cognitive domains and constitute a protective factor against brain damage or aging.

## Ethics Statement

Healthy young participants gave their written informed consent prior to taking part to the study. The research protocol was approved by the CPP Nord-Ouest ethic committee.

## Author Contributions

HP and MtG, designed and developed the study. PG, BF, and MlG analyzed the data. PG, BF, MtG, and HP wrote the manuscript. BD, FV, and FE contributed to theoretical and methodological refinements and provided detailed suggestions for manuscript revisions.

## Conflict of Interest Statement

The authors declare that the research was conducted in the absence of any commercial or financial relationships that could be construed as a potential conflict of interest.
