# Supplementary material for: Musical Expertise Increases Top–Down Modulation Over Hippocampal Activation during Familiarity Decisions
Source: Front Hum Neurosci. 2017 Sep 26;11:472. doi: 10.3389/fnhum.2017.00472 (PMC5626938; doi:10.3389/fnhum.2017.00472)
Supplement: Supplementary file 1 [file Table_1.docx]

***Table S1:*** *Peak coordinates and statistics of regions showing a main effect of familiarity for each type of material, as well as a Material x Familiarity interaction at p*_FWE_ *<* *.05, for a minimum cluster size of k = 10*. Distinct local maxima within the same cluster are shown in italics.

| Contrast and anatomical labeling | Number of voxels | MNI coordinates (mm) | | | *T* statistics | *p*_FWE_ values |
| --- | --- | --- | --- | --- | --- | --- |
|  |  | x | Y | z |  |  |
| 1. *Main effect of familiarity* | | | | | | |
| *1.a Familiar versus unfamiliar items* | | | | | | |
| Left postcentral gyrus | 40506* | -42 | -20 | 58 | 28.70 | <.001 |
| *Right culmen of the cerebellum* |  | *20* | *-52* | *-22* | *21.26* | *<.001* |
| *Left lentiform nucleus (lateral globus pallidus)* |  | *-28* | *-14* | *-4* | *13.45* | *<.001* |
| *Left cingulate gyrus* |  | *-4* | *-22* | *46* | *12.64* | *<.001* |
| *Left superior parietal lobule* |  | *-22* | *-40* | *68* | *12.32* | *<.001* |
| *Left thalamus* |  | *-4* | *-6* | *10* | *11.39* | *<.001* |
| *Left inferior frontal gyrus* |  | *-34* | *30* | *-20* | *10.45* | *<.001* |
| *Left superior frontal gyrus* |  | *-10* | *66* | *12* | *10.01* | *<.001* |
| *Left precentral gyrus* |  | *-16* | *-12* | *70* | *9.5* | *<.001* |
| Right inferior parietal lobule | 612 | 54 | -42 | 50 | 9.18 | <.001 |
| *Right supramarginal gyrus* |  | *64* | *-50* | *32* | *5.69* | *<.01* |
| *Right superior parietal lobule* |  | *40* | *-64* | *50* | *5.4* | *<.05* |
| Left inferior temporal gyrus | 150 | -56 | -50 | -10 | 7.9 | <.001 |
| *Left middle temporal gyrus* |  | *-58* | *-34* | *-10* | *5.71* | *<.01* |
| Right inferior frontal gyrus | 218 | 30 | 32 | -14 | 7.58 | <.001 |
| *Right claustrum* |  | *30* | *16* | *-16* | *6.05* | *<.001* |
| Right middle frontal gyrus | 213 | 32 | 22 | 48 | 6.38 | <.001 |
|  |  | *40* | *8* | *54* | *5.65* | *<.01* |
| Left inferior temporal gyrus | 51 | -60 | -14 | -18 | 6.13 | <.01 |
| Right lingual gyrus | 20 | 24 | -60 | 2 | 5.47 | <.01 |
| Right parahippocampal gyrus | 51 | 24 | -20 | -12 | 5.43 | <.01 |
| *Right lentiform nucleus (lateral globus pallidus)* |  | *30* | *-12* | *-12* | *6.04* | *<.05* |
| Right superior temporal gyrus | 14 | 54 | -26 | -12 | 5.3 | <.05 |
| Right middle frontal gyrus | 10 | 52 | 40 | 14 | 5.17 | <.05 |
| *1.b Unfamiliar versus familiar items* | | | | | | |
| Right precentral gyrus | 3891 | 42 | -20 | 58 | 28.26 | <.001 |
| *Right superior parietal lobule* |  | *28* | *-52* | *68* | *9.21* | *<.001* |
| Left culmen of the cerebellum | 616 | -16 | -50 | -20 | 18.31 | <.001 |
| Right insula | 609 | 46 | -16 | 18 | 14.15 | <.001 |
| *Right lentiform nucleus (putamen)* |  | *32* | *-6* | *-2* | *5.84* | *<.01* |
| Right medial frontal gyrus | 224 | 8 | -10 | 52 | 9.02 | <.001 |
| *Right paracentral lobule* |  | *10* | *-20* | *52* | *8.07* | *<.001* |
| Right thalamus | 21 | 16 | -20 | 4 | 6.17 | <.001 |
| 1. *Familiarity*material* | | | | | | |
| *2.a [(familiar melodies > unfamiliar melodies) versus (familiar proverbs > unfamiliar proverbs)]* | | | | | | |
| Left superior frontal gyrus | 1269 | -2 | 12 | 58 | 8.43 | <.001 |
| *Right medial frontal gyrus* |  | *6* | *16* | *42* | *7.16* | *<.001* |
| *Left cingulate gyrus* |  | *-4* | *14* | *42* | *6.82* | *<.001* |
| Right inferior frontal gyrus | 805 | 32 | 32 | -12 | 8.08 | <.001 |
| *Right insula* |  | *36* | *24* | *-4* | *7.01* | *<.001* |
|  |  | *44* | *26* | *8* | *6.26* | *<.001* |
| Left middle frontal gyrus | 1897 | -40 | 16 | 24 | 7.71 | <.001 |
| *Left insula* |  | *-34* | *26* | *-2* | *7.49* | *<.001* |
| *Left inferior frontal gyrus* |  | *-32* | *32* | *-12* | *6.89* | *<.001* |
| Left caudate body | 595 | -10 | 4 | 6 | 7.53 | <.001 |
| *Left lentiform nucleus (putamen)* |  | *-16* | *10* | *-2* | *6.76* | *<.001* |
| *Left caudate body* |  | *-10* | *2* | *14* | *6.27* | *<.001* |
| Right caudate head | 557 | 14 | 12 | -4 | 7.44 | <.001 |
| *Right caudate body* |  | *16* | *2* | *12* | *5.95* | *<.01* |
| *Right lentiform nucleus (medial globus pallidus)* |  | *18* | *0* | *-6* | *5.78* | *<.01* |
| Left parahippocampal gyrus | 76 | -26 | -32 | -18 | 6.76 | <.001 |
| Left substania nigra | 176 | -8 | -26 | -12 | 6.44 | <.001 |
| *Right thalamus* |  | *4* | *-28* | *0* | *5.45* | *<.01* |
| Left lentiform nucleus (medial globus pallidus) extending to the left hippocampus | 32 | -14 | -4 | -12 | 5.57 | <.01 |
| *2.b [(familiar proverbs > unfamiliar proverbs) versus[(familiar melodies > unfamiliar melodies)]* | | | | | | |
| Left inferior occipital gyrus | 247 | -44 | -80 | 0 | 6.97 | <.001 |
| *Left fusiform gyrus* |  | *-28* | *-78* | *-10* | *6* | *<.01* |
| *Left declive of the cerebellum* |  | *-38* | *-80* | *-12* | *5.16* | *<.05* |

**Note:* Given the extremely large size of this cluster, we only report the most substantial local maxima.
